# Intraocular siRNA Delivery Mediated by Penetratin Derivative to Silence Orthotopic Retinoblastoma Gene

**DOI:** 10.3390/pharmaceutics15030745

**Published:** 2023-02-23

**Authors:** Xin Gao, Xingyan Fan, Kuan Jiang, Yang Hu, Yu Liu, Weiyue Lu, Gang Wei

**Affiliations:** 1 Key Laboratory of Smart Drug Delivery, Ministry of Education & Department of Pharmaceutics, School of Pharmacy, Fudan University, Shanghai 201203, China; 2 Department of Pharmacology, School of Basic Medical Sciences & State Key Laboratory of Molecular Engineering of Polymers, Fudan University, Shanghai 200032, China; 3 The Institutes of Integrative Medicine of Fudan University, Shanghai 200040, China; 4 Shanghai Engineering Research Center of ImmunoTherapeutics, Shanghai 201203, China

**Keywords:** gene delivery, intraocular drug delivery, siRNA, penetratin derivative, nonviral vector, noninvasive administration

## Abstract

Gene therapy brings a ray of hope for inherited ocular diseases that may cause severe vision loss and even blindness. However, due to the dynamic and static absorption barriers, it is challenging to deliver genes to the posterior segment of the eye by topical instillation. To circumvent this limitation, we developed a penetratin derivative (89WP)-modified polyamidoamine polyplex to deliver small interference RNA (siRNA) via eye drops to achieve effective gene silencing in orthotopic retinoblastoma. The polyplex could be spontaneously assembled through electrostatic and hydrophobic interactions, as demonstrated by isothermal titration calorimetry, and enter cells intactly. In vitro cellular internalization revealed that the polyplex possessed higher permeability and safety than the lipoplex composed of commercial cationic liposomes. After the polyplex was instilled in the conjunctival sac of the mice, the distribution of siRNA in the fundus oculi was significantly increased, and the bioluminescence from orthotopic retinoblastoma was effectively inhibited. In this work, an evolved cell-penetrating peptide was employed to modify the siRNA vector in a simple and effective way, and the formed polyplex interfered with intraocular protein expression successfully via noninvasive administration, which showed a promising prospect for gene therapy for inherited ocular diseases.

## 1. Introduction

According to a recent epidemiological survey, nearly 295 million people worldwide suffer from moderate to severe visual impairments, which cause about 43.3 million blindness [1]. Even worse, some inherited blinding diseases, for example X-linked retinoschisis and Leber congenital amaurosis, still lack effective treatment regimens. Fortunately, gene therapy brings a ray of hope for these diseases [2]. The regulation of gene expression by nucleic acid agents has become one of the most promising strategies for the treatment of inherited blinding diseases, and more than 40 kinds of gene therapeutics are currently under clinical evaluation [3].

With its unique anatomy and immune-privilege characteristics, the eye is an ideal organ for topical administration and gene therapy [4,5]. Eye drops are convenient and can avoid the interference of a complicated physiological environment on gene delivery, as encountered by systemic administration. However, due to the dynamic and static absorption barriers of the eye, nucleic acid agents are obliged to be applied via subretinal or intravitreal injection, which requires rigorous medical conditions and is accompanied by poor patient compliance [6,7]. In late 2017, voretigene neparvovec-rzyl (Luxturna), a breakthrough in gene therapy, was approved by the U.S. Food and Drug Administration for the treatment of inherited retinal dystrophy. The human retinal pigment epithelial 65 kDa protein (*hRPE65*) genome is successfully encoded into the recombinant adenovirus-associated viruses (rAAV) vector, and then injected into the retina of patients to enable gene expression. This would allow the normally functioning photoreceptors to survive, and thus restore visual perception [2,8]. Although gene regulation provides a substantial therapeutic advance, intraocular injection may cause various side effects with an incidence higher than 5%, and even permanent vision loss [8]. Therefore, noninvasive delivery via topical instillation has great potential to meet the urgent clinical needs for the development of gene therapy [9].

Gene delivery is typically mediated by viral or nonviral vectors [10]. Viral vectors possess the advantages of high transfection efficiency, easy purification, and a strong ability to combine with genes [11]. However, they are usually afflicted with potential immunotoxicity [12], insertional mutagenesis, and limited loading capability, which hinders them from broadly clinical application [13]. On the contrary, the most prominent benefit of nonviral vectors is that they can alleviate the immune deficiency of viral vectors and regulate the loading capability of genes [14]. More importantly, nonviral vectors could be applied for noninvasive administration, making delivery more flexible and feasible, convenient, and safe [15]. However, the transfection efficiency of nonviral vectors is relatively low [16]. Thus, improvement of the ability of nonviral vectors to compress, deliver, and transfect genes via a simple and effective approach would be immensely beneficial to the clinical translation of gene therapy.

Retinoblastoma is an intraocular malignancy mostly affecting infants and children caused by hereditary gene mutations. Current clinical interventions include intravenous, intraarterial, or intravitreal chemotherapy, surgeries, and radiotherapy [17]. Some attempts have also been made to apply nano-drug delivery systems loaded with cytotoxic anti-tumor drugs or genes to treat retinoblastoma [18]. In the present work, we aimed to develop a nonviral vector that would deliver small interference RNA (siRNA) into the eye via topical instillation to manage retinoblastoma. siRNAs are characterized by high molecular weight, abundant negative charges, strong hydrophilicity, and poor stability in vitro and in vivo [19], so a variety of cationic polymers that can compress and neutralize genes are widely utilized to form polyplexes with siRNA through electrostatic interactions [20]. Polyamidoamine (PAMAM) dendrimer is one of the most commonly used gene carriers [21] due to its unique three-dimensional configuration, good biocompatibility, permeability, and stability. The 3rd generation PAMAM (PA) was chosen to pre-compress siRNA herein because of its low toxicity, but correspondingly, its delivery efficacy is relatively weak. Previously, we found that based on wild-type cell-penetrating peptide (CPP) penetratin, more powerful absorption enhancers, for example Q8W and N9W-penetratin (89WP), could be derived for ocular delivery [22]. Hence, 89WP was further employed to modify the primary polyplex composed of siRNA and 3rd generation PAMAM (siRNA/PA), forming a topical delivery system siRNA/PA/89WP to improve absorption of siRNA in the posterior segment of the eye. We recently used wild-type penetratin and 5th generation PAMAM to compress antisense oligonucleotides (ASOs) [23]. In order to modify PAMAM with the positively charged penetratin, negatively charged hyaluronic acid had to be introduced into the complex to implement layer-by-layer self-assembly via electrostatic interaction [24]. In this study, we tried to develop a simpler polyplex by direct modification with the evolved penetratin derivative to achieve more efficient intraocular delivery of siRNA. We hypothesized that 89WP was able to self-assemble with the siRNA/PA polyplex without the involvement of negatively charged hyaluronic acid. The assembling mechanism of this siRNA delivery system was investigated, and the efficacy of gene interference was compared with the commercially available transfection agent Lipofectamine 2000 (Lipo) using an orthotopic retinoblastoma model.

## 2. Materials and Methods

### 2.1. Materials

Small interference RNA targeting the exogenetic luciferase gene, whose sense sequence is 5’-GCACUCUGAUUGACAAAUATT-3’ and antisense sequence is 5’-UAUUUGUCAAUCAGAGUGCTT-3’, was synthesized by GenePharma (Jiangsu, China). The 5’ terminus of siRNA was labeled with 5-carboxyfluorescein (siRNA-FAM) and Cy5 (siRNA-Cy5) in the respective experiment. The penetratin-derived peptide 89WP and 5-carboxyfluorescein-labeled 89WP (89WP-FAM) were synthesized by China Peptides (Shanghai, China), whose amino acid sequence was RQIKIWFWWRRMKWKK. The 3rd generation PAMAM (MW 6.9 kDa) was purchased from Sigma-Aldrich (St. Louis, MO, USA). Lipofectamine 2000 (Lipo, Sydney, Australia) was obtained from Thermo Fisher Scientific (Waltham, MA, USA), and luciferin, the substrate of luciferase, was purchased from Sciencelight (Shanghai, China). All other chemicals used were of analytic grade.

### 2.2. Cell Lines and Animals

Human retinal glial cells (WERI-Rb-1) and retinal pigment epithelium cells (ARPE-19) were provided by the Cell Bank, Chinese Academy of Science (Shanghai, China). WERI-Rb-1-luci cells that express luciferase were built using lentiviral vectors encoding firefly luciferase (luci) to infect WERI-Rb-1 cells [25]. RPMI 1640 Dulbecco’s modified Eagle medium containing nutrient mixture F-12 (DMEM), fetal bovine serum (FBS), 0.25% trypsin-EDTA, and penicillin-streptomycin was obtained from Gibco (Carlsbad, CA, USA). Cell counting kit-8 (CCK-8) was purchased from Meilun (Dalian, China). Cell culture plates and flasks were purchased from Cellvis (Mountain View, CA, USA).

Male nude mice (18–20 g) and male ICR mice (18–20 g) were purchased from the Experimental Animal Center of Fudan University and kept under specified conditions. All animal experiments were performed in accordance with the protocols and guidelines approved by the Ethics Committee of Fudan University (2021-06-YL-WG-81). Animals were acclimatized to laboratory conditions for 1 week before the experiments.

### 2.3. Preparation of siRNA Polyplexes

Preparation of the polyplex siRNA/PA/89WP involved two steps. First, the primary polyplex siRNA/PA was prepared by mixing an equal volume of 130 μg/mL PAMAM solution with 100 μg/mL siRNA solution, followed by vortexing for 30 s and then incubation for 30 min under static conditions. The ratio of nitrogen in PAMAM to phosphorus in the siRNA (N/P) of the primary polyplex in the mixed solution was 2:1, which was optimized by polyacrylamide gel electrophoresis. Second, an equal volume of 89WP solution was added to the siRNA/PA solution at different charge ratios of 89WP to siRNA (5:1, 8:1, 10:1, 15:1, 20:1, and 30:1), followed by vortex and incubation, as described above.

For preparation of the lipoplex siRNA/Lipo, an aliquot of 300 μg/mL siRNA solution was mixed with an equal volume of Lipofectamine 2000 solution according to the instructions, followed by vortexing for 30 s and then incubation for 30 min. All the above operations were performed at room temperature.

### 2.4. Characterization of siRNA Polyplexes

The particle size and zeta potential of the polyplexes were measured by dynamic light scattering (DLS, Malvern Instruments, Malvern, UK) at room temperature. The size distribution was presented by intensity. Each sample was replicated for 3 times. The refractive index was 1.59, and the detector angle was 173°. The morphology of the polyplexes was observed under a transmission electron microscope (FEI, Hillsboro, OR, USA). Briefly, 5 µL of each sample was added onto glow-discharged carbon-coated grids for 5 min, and then the remaining liquid was dried in an oven at 37 °C. The samples were visualized under a microscope operating at an accelerating voltage of 200 keV in bright-field image mode.

### 2.5. Cellular Uptake

In qualitative observation, the ARPE-19 cells in the logarithmic growth phase were inoculated in 24-well plates with 20,000 cells in each well (*n* = 3) and incubated for 24 h with 5% CO_2_ at 37 °C in a Heraeus incubator (Kendro, San Francisco, CA, USA). The cells were then cultured in serum-free medium with various formulations at a final concentration of 1 µg/mL siRNA. After 4 h incubation, the cells were washed with 0.2% (*m*/*v*) heparin sodium solution three times, fixed with 4% (*m*/*v*) paraformaldehyde solution for 5 min, and then moisturized with 50% (*v*/*v*) glycerol. After that, the cells were then observed under an inverted fluorescence microscope (DMI4000B, LEICA, Germany). FAM fluorescence was excited at a 488 nm wavelength using an argon laser, and the emission was detected at 520 nm.

For quantitative analysis of cellular uptake of different formulations, ARPE-19 cells and WERI-Rb-1 cells in the logarithmic growth phase were inoculated in 12-well plates with 50,000 cells in each well (*n* = 3), as mentioned above. After being incubated with various formulations at a final concentration of 1 µg/mL siRNA, the cells were trypsinized with 0.25% trypsin-EDTA, collected in Eppendorf tubes, and centrifuged at 1000 rpm for 5 min. The precipitated cells were subsequently suspended, washed twice in 200 μL phosphate buffer saline (PBS), and analyzed using a flow cytometer (Beckman, Brea, CA, USA). The percentage of FAM-positive cells in total viable cells was defined as the uptake efficiency. The effects of various inhibitors on cellular uptake were also assessed. The applied concentrations and function mechanisms of the inhibitors are listed in Table S1.

### 2.6. In Vitro Gene Silencing

WERI-Rb-1-luci cells, which express luciferase in logarithmic growth phase and good condition, were inoculated in 24-well plates with 100,000 cells in each well (*n* = 3) and incubated for 24 h with 5% CO_2_ at 37 °C. The cells were then administered various polyplexes at a final concentration of 1 µg/mL siRNA and incubated in serum-free medium for 4 h. After further cultivation in complete medium for 20 h, an equal volume of 0.15 mg/mL luciferin solution was added to each well for bioluminescence observation under an IVIS Spectrum system (Cailper PerkinElemer, Waltham, MA, USA).

### 2.7. Cell Viability Assay

ARPE-19 cells and WERI-Rb-1 cells in logarithmic growth phase and good condition were inoculated in 96-well plates at a density of 5000 cells per well (*n* = 3), and the edges of the plates were filled with sterile PBS. Following incubation at 37 °C and 5% CO_2_ for 24 h, various polyplex formulations were added at a final concentration of 1 µg/mL siRNA and further incubated for 6 h. Afterwards, 10 µL CCK-8 solution was added to each well directly. The cells were incubated for an additional 2 h, and the absorbance value of each well was measured on a micro-plate reader (Bio-Tek, Shoreline, WA, USA) at a detection wavelength of 450 nm. The percentage of cell *viability* was calculated compared with that of untreated cells.

### 2.8. Isothermal Titration Calorimetry

An aliquot of 200 μL siRNA solution (70 µg/mL) or primary polyplex siRNA/PA solution (160 µg/mL) was filled into the sample cell of isothermal titration calorimeter (MicroCal iTC200, GE, USA), and about 40 μL of PA solution (240 µg/mL) or 89WP solution (2.80 mg/mL) was inhaled into the titration injector, respectively. The duration of each injection was 4 s, and the interval between each injection was 150 s. In order to ensure complete mixing in a few seconds, the injector stirred the solution in the sample cell at a rate of 1000 rpm. The system temperature was set at 25 °C. Titration was carried out first using a sample solution and then pure water as a control. Calorimetric data were analyzed using Origin software.

### 2.9. Intracellular Co-Localization

ARPE-19 cells in the logarithmic growth phase were seeded in confocal dishes with a density of 5000 cells/well and incubated for 24 h with 5% CO_2_ at 37 °C. After administration with various polyplexes at a final concentration of 1 µg/mL siRNA, the cells were further cultured in serum-free medium for 4 h. Then, the cells were washed with 0.2% (*m*/*v*) heparin sodium solution three times, fixed with 4% (*m*/*v*) paraformaldehyde solution for 5 min, stained with 1 µg/mL 4’,6-diamidino-2-phenylindole (DAPI) solution for 10 min, and moisturized with 50% (*v*/*v*) glycerol. After that, the cells were observed with a laser scanning confocal microscope (Carl Zeiss, Jena, Germany).

### 2.10. In Vivo Retinal Distribution

Twenty mice were randomly divided into four groups, namely control group, naked siRNA group, polyplex siRNA/PA/89WP group, and lipoplex siRNA/Lipo group. In the control group (*n* = 2), 5 µL normal saline was instilled into the conjunctival sac of the right eyes of mice, while in the treatment groups (*n* = 6), the same volume of various formulations containing 2 µg siRNA labeled with FAM was applied. After topical administration, mice were sacrificed at 0.5, 1, 2, 4, 6, and 8 h by injection with a lethal dose of pentobarbital sodium (150 mg/kg). The eyeballs were harvested and fixed overnight with 4% (*m*/*v*) paraformaldehyde PBS solution, followed by preparation of frozen slices, which were observed with a laser scanning confocal microscope (Carl Zeiss, Germany) and analyzed using ZEN software.

### 2.11. In Vivo Gene Silencing

Nude mice were anesthetized by intraperitoneal injection with 40 mg/kg pentobarbital sodium solution. The right eye of each mouse was inoculated with 20,000 WERI-Rb-1-luci cells in the logarithmic growth phase by intravitreal injection, and the left eye was set as the control. The bioluminescence intensity of orthotopic retinoblastoma was observed regularly. The day of initial tumorigenesis (the 7th day after inoculation) was recorded as day 0, and on the same day, administration was also started.

Twelve nude mice were randomly divided into 4 groups, namely control group, naked siRNA group, siRNA/PA/89WP group, and siRNA/Lipo group. From day 0, the mice were given 5 µL various formulations containing 1.5 µg siRNA, except for the control group given normal saline, by topical instillation 3 times a day for 15 consecutive days.

On days 0, 3, 5, 7, 9, 11, 13, and 15, the bioluminescence of the inoculated eyes was determined. After intraperitoneal injection with 150 mg/kg luciferin, each mouse was anesthetized with isoflurane and subjected to in vivo bioluminescence imaging. The bioluminescence intensity of the retinoblastoma was semi-quantified to observe the variation. On day 15, after the last bioluminescence determination, all mice were sacrificed, and their right eyeballs were harvested for hematoxylin-eosin staining to observe the integrity of the cornea.

### 2.12. Statistical Analysis

The statistical significance of the quantitative data was analyzed via multiple comparisons of one-way or two-way ANOVA or *t*-test corrected by GraphPad Prism software. One asterisk (*) represents a significant difference (*p* < 0.05), two (**), three (***), and four (****) asterisks represent a highly significant difference (*p* < 0.01, *p* < 0.001, and *p* < 0.0001, respectively) and ns represents no significant difference (*p* > 0.05).

## 3. Results

### 3.1. Formation and Characterization of siRNA Polyplexes

**Figure 1:**
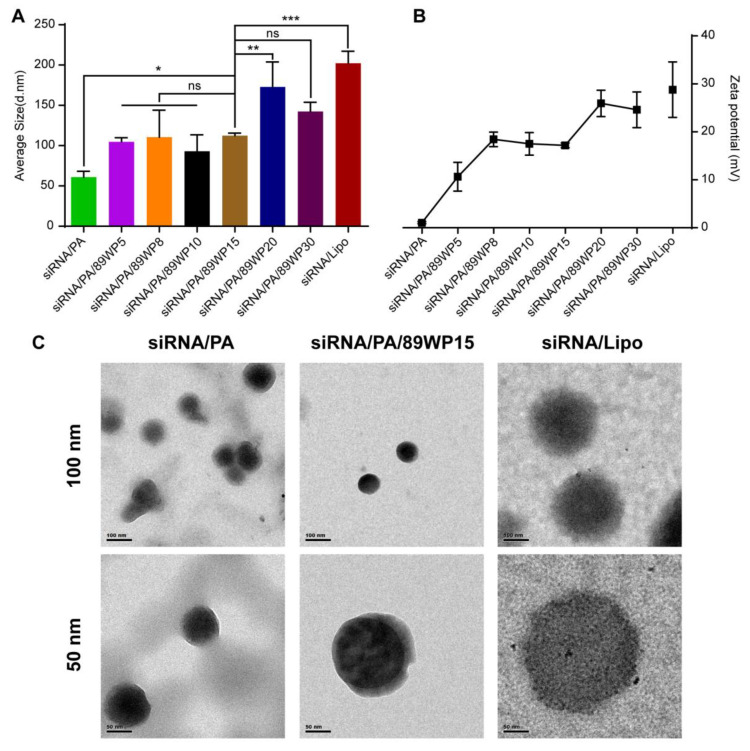
Characterization of siRNA polyplexes. (**A**) The particle sizes of various polyplex formulations measured using DLS. (**B**) Zeta potential of various polyplex formulations. (**C**) Morphology of the polyplexes siRNA/PA, siRNA/PA/P15, and lipoplex siRNA/Lipo observed by TEM. The scale bars are 100 nm for the first row and 50 nm for the second. Data are presented as mean ± SD (*n* = 3).

As seen from the polyacrylamide gel electrophoresis (Figure S1), when the N/P ratio of 3rd generation PAMAM to siRNA was higher than 2:1, PAMAM could basically compress the siRNA and shield the negative charge. Therefore, in the primary polyplex siRNA/PA, the N/P ratio was chosen as 2:1. Then, 89WP was added to the primary polyplex at different ratios, forming siRNA/PA/89WPn, where n represents the charge ratio of 89WP to siRNA. As shown in Figure 1A,B, the primary polyplex siRNA/PA had a particle size of about 60 nm and exhibited electric neutrality. When 89WP was gradually added, both the particle size and zeta potential showed a tendency to increase, but they were lower than those of the lipoplex siRNA/Lipo (about 200 nm and 30 mV, respectively). The fluctuation in particle sizes of different polyplex formulations was mainly affected by two factors: the compression efficiency of siRNA and the composition of the polyplex. With the increase in the charge ratios, more peptide molecules were introduced into the polyplexes to improve the compression of siRNA. Simultaneously, these peptide molecules involved in the formation of the polyplexes also led to a slight increase in particle size. A smaller size means easier internalization by cells [26], and lower zeta potential implies better biocompatibility than siRNA/Lipo. The morphology of polyplexes siRNA/PA, siRNA/PA/P15, and lipoplex siRNA/Lipo observed by TEM was presented as a solid spherical shape, revealing that PAMAM and 89WP could compress siRNA tightly (Figure 1C). The particle sizes observed by TEM were consistent with those measured by DLS, as shown in Figure 1A. The particle size of polyplex siRNA/PA/P15 remained stable at 4 °C for at least 48 h (Figure S2).

### 3.2. Optimization of the Ratio of 89WP to siRNA

**Figure 2:**
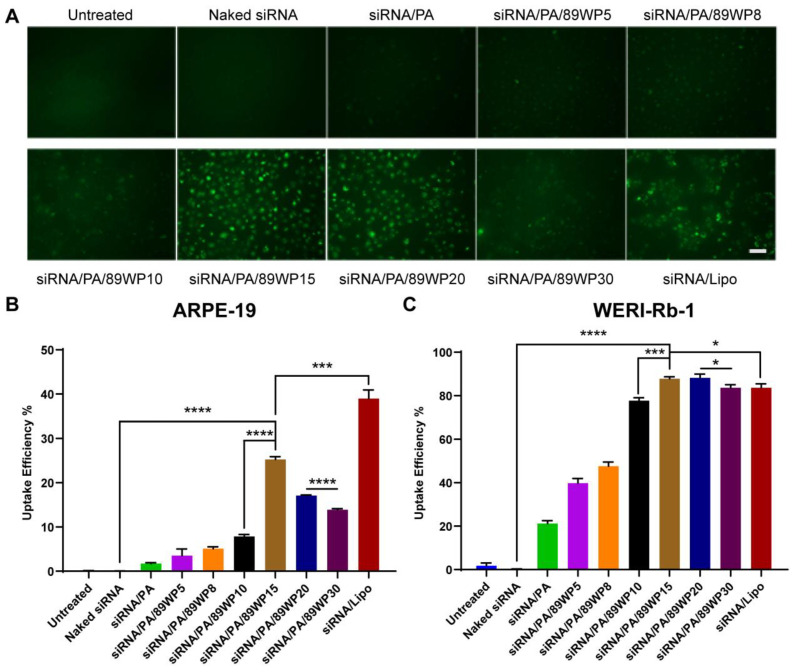
Optimization of the ratio of 89WP to siRNA via qualitative observation and quantitative flow cytometry evaluation. (**A**) The uptake of various siRNA polyplex formulations in ARPE-19 cells was qualitatively observed using an inverted fluorescence microscope (Scale bar, 100 µm). Flow cytometry was used to quantitatively investigate the uptake percentage of the siRNA polyplexes at different charge ratios of 89WP to siRNA in ARPE-19 (**B**) and WERI-Rb-1 (**C**) cells. The numbers behind 89WP in each polyplex formulation represent the charge ratios of 89WP to siRNA. Data are presented as mean ± SD (*n* = 3).

Cellular uptake of various formulations was evaluated via qualitative observation using an inverted fluorescence microscope (Figure 2A). It was found that the fluorescence of siRNA-FAM in the co-incubated ARPE-19 cells increased gradually with the proportion of 89WP, and when the charge ratio of 89WP to siRNA ranged between 15:1 and 20:1, the highest cellular uptake occurred. For the cells treated by siRNA/Lipo prepared according to the instructions of Lipofectamine 2000, the fluorescence was almost as bright as the polyplexes containing 89WP, but the cells adhered to each other and looked in poor condition.

Flow cytometry was used to quantitatively investigate the uptake efficiency of siRNA polyplexes in normal ARPE-19 cells and tumor WERI-Rb-1 cells. The naked siRNA could barely be internalized alone by both cells (Figure 2B,C) due to its large molecular weight, strong hydrophilicity, and electronegativity. After being compressed by PAMAM, the uptake efficiencies of the primary polyplex siRNA/PA in ARPE-19 and WERI-Rb-1 cells were about 3% and 20%, respectively, which were substantially improved compared to the naked siRNA. With the addition of the peptide 89WP, the uptake efficiency in these two cell lines further increased with the proportion of 89WP, and peaked when the charge ratio of 89WP to siRNA reached 15:1. The uptake efficiency of siRNA/PA/89WP15 in ARPE-19 cells was 8.3 times higher than that of primary polyplex siRNA/PA (Figure 2B). Particularly in WERI-Rb-1 cells, the uptake efficiency of siRNA/PA/89WP15 reached 90%, which was 4.5 times higher than that of primary polyplex siRNA/PA, and was also significantly higher than that of siRNA/Lipo (Figure 2C), indicating that 89WP could greatly promote siRNA internalization. When the charge ratio of 89WP to siRNA further increased, the quantitative cellular uptake trended to decrease slightly, which might reflect the potential cytotoxicity caused by the polyplex formulations. It is worth noting that the uptake efficiencies of all the polyplexes and lipoplexes in the WERI-Rb-1 cells were much higher than in the ARPE-19 cells. We speculate that these differences were mainly due to the inherent endocytic capability of various cell lines to nanoparticles. This result is consistent with a previous report by Patiño et al. [27], who found that tumoral human breast epithelial cells (SKBR-3) mainly internalized positively charged microparticles, with a 3-fold higher efficiency than normal human breast epithelial cells (MCF-10A). In contrast, an opposed cellular uptake effect was observed for the negatively charged microparticles. The authors attributed the completely different endocytic capabilities of the two cell lines to their tumorigenic or non-tumorigenic nature. Since our polyplexes were all positively charged, they were more apt to be endocytosed by tumor cell WERI-Rb-1.

**Figure 3:**
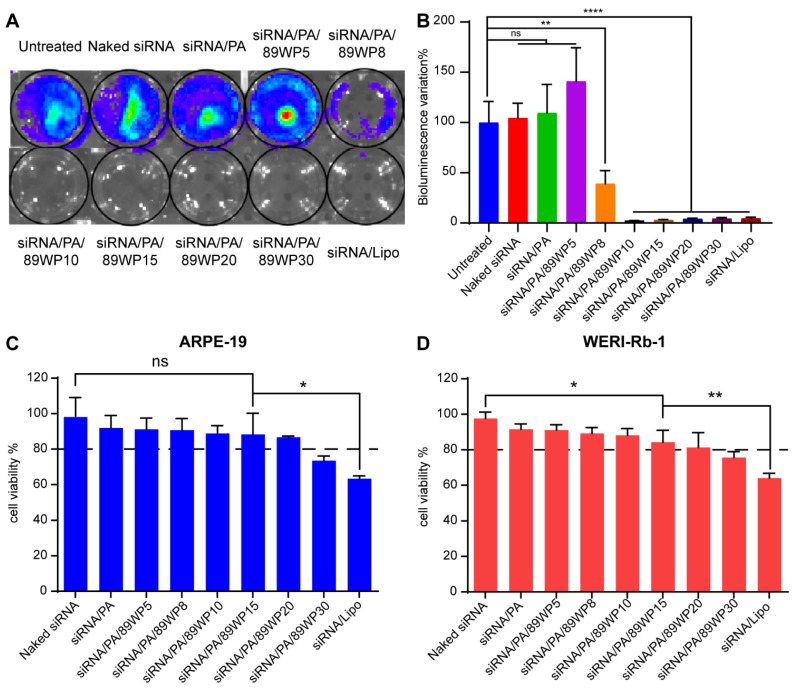
Optimization of the ratio of 89WP to siRNA by in vitro gene silencing effect and cell viability. Bioluminescence imaging was conducted to (**A**) qualitatively and (**B**) semi-quantitatively evaluate the interference effect of luciferase expression in WERI-Rb-1-luci cells using different siRNA polyplex formulations. Data were presented as mean ± SD (*n* = 4), and significance was compared with the untreated group. CCK-8 was used to evaluate the cell viability of different formulations to (**C**) ARPE-19 and (**D**) WERI-Rb-1 cells. Data were presented as mean ± SD (*n* = 3), and significance was compared with siRNA/PA/89WP15 group.

Bioluminescence imaging was conducted for qualitative (Figure 3A) and semi-quantitative (Figure 3B) evaluations of the interference effects on luciferase expression in WERI-Rb-1-luci cells using various siRNA polyplexes. No gene interference was observed in the cells treated with naked siRNA or the primary polyplex siRNA/PA. When the charge ratio of 89WP to siRNA reached 10:1, the expression of luciferase was basically inhibited (Figure 3B), which further proved the absorption-enhancing effect of 89WP.

CCK-8 was used to investigate the toxicity of various formulations to ARPE-19 and WERI-Rb-1 cells (Figure 3C,D). It was found that when the charge ratio of 89WP to siRNA was lower than 20:1, the survival rate of both cells was higher than 80%, and the biosafety of these formulations was acceptable. In the normal cell ARPE-19, there was no significant difference in the viabilities between the cells treated with polyplex siRNA/PA/89WP15 and the negative control naked siRNA, indicating that the carrier materials 3rd generation PAMAM and 89WP at this dose did not cause significant toxicity to the cells. In contrast, the positive control siRNA/Lipo was highly toxic to both cells, which reduced cell viability to below 70%, and the toxicity was significantly higher than that of siRNA/PA/89WP15. Considering the internalization, interference effect, and toxicity, siRNA/PA/89WP15 was chosen as the final polyplex formulation, also known as siRNA/PA/89WP hereafter.

### 3.3. Interactions between Components of siRNA Polyplexes

**Figure 4:**
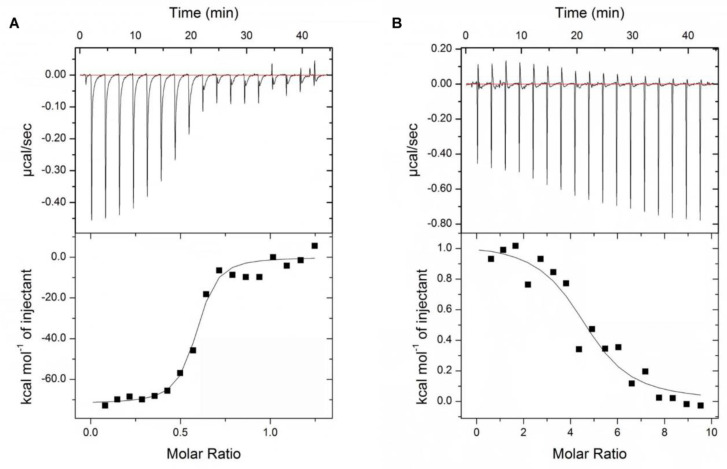
Interactions between the components of siRNA polyplexes. Isothermal titration curves for the interaction between siRNA and PAMAM (**A**), and for the interaction between 89WP and siRNA/PA (**B**).

Isothermal titration calorimetry (ITC) was implemented to determine the interactions between PAMAM and siRNA and between 89WP and primary polyplex siRNA/PA. The ITC titration curves are shown in Figure 4, and the fitted thermodynamic parameters are presented in Table 1. When PAMAM was mixed with siRNA, the equilibrium binding constant (K_b_) value was 3.63 ± 1.25 × 10^7^ M^−1^, accompanied by an exothermic process (∆H < 0) and reduced entropy (∆S < 0). The corresponding Gibbs free energy change (∆G), calculated via the equation ∆G = ∆H − T∆S, was also negative. Because under neutral pH, PA is positively charged, while siRNA is negatively charged, they could form stable primary polyplexes under room temperature due to electrostatic interaction. The thermodynamic parameters revealed that the mixed system became more orderly and that the primary polyplexes were spontaneously formed.

However, the ITC titration curve of 89WP and siRNA/PA in Figure 4B showed a completely different tendency from that of PA and siRNA as shown in Figure 4A. According to the corresponding thermodynamic parameters in Table 1, the K_b_ value was 1.85 ± 0.90 × 10^5^ M^−1^, which was two orders of magnitude lower than that calculated from PA and siRNA, but there was still interaction between 89WP and siRNA/PA. The mixing process of 89WP and siRNA/PA was endothermic (∆H > 0) with increased entropy change (∆S > 0), suggesting that in addition to the formation of a polyplex, there might be some free 89WP in the system. The calculated Gibbs free energy change was also negative (∆G < 0) and had a similar value to that obtained during formation of the primary polyplex siRNA/PA. These results indicated that 89WP and siRNA/PA could spontaneously form stable polyplexes at room temperature, perhaps via an entropy-driven process characterized by hydrophobic interaction [28].

### 3.4. Integrity of siRNA Polyplexes during Cellular Uptake

**Figure 5:**
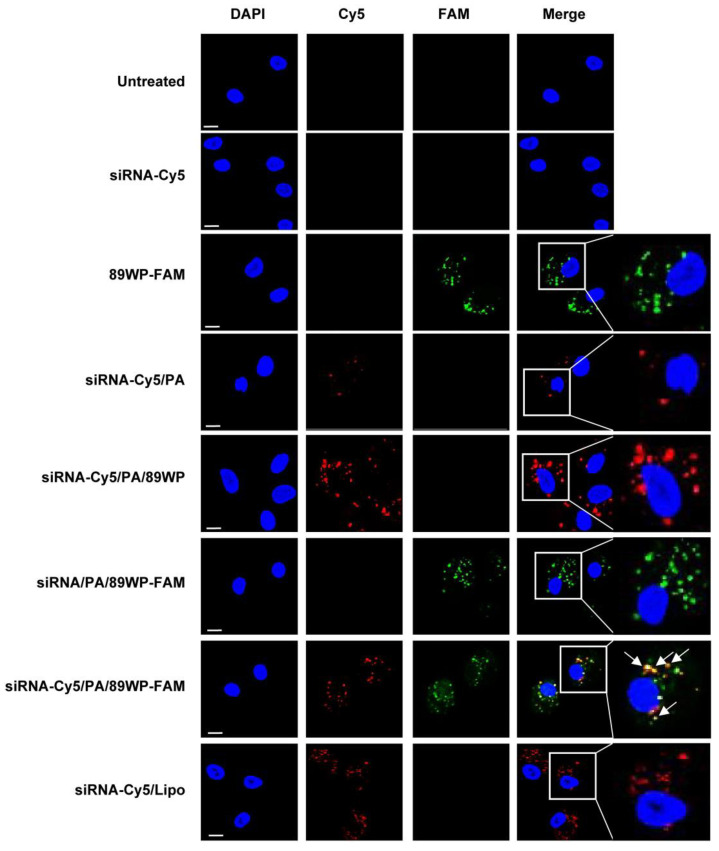
Co-localization of the polyplex components in cells. The siRNA labeled with Cy5 (siRNA-Cy5, red) and 89WP labeled with FAM (89WP-FAM, green) formed polyplexes siRNA-Cy5/PA/89WP, siRNA/PA/89WP-FAM, siRNA-Cy5/PA/89WP-FAM, and lipoplex siRNA-Cy5/Lipo. The distribution and co-localization (yellow) of siRNA-Cy5 and 89WP-FAM in ARPE-19 cells were observed under a confocal microscope. Scale bar, 20 µm.

Laser confocal microscopy was used to investigate the co-localization of siRNA and 89WP after the polyplexes were internalized by ARPE-19 cells. As shown in Figure 5, the cell nuclei stained with DAPI are blue, the siRNA labeled with Cy5 is red, and the 89WP labeled with FAM is green. Naked siRNA-Cy5 could not enter the cells; however, when compressed by PAMAM and forming primary polyplex siRNA-Cy5/PA, a low level of internalization could be observed. These results are consistent with observations in cellular uptake studies. In contrast, the peptide 89WP-FAM could be well internalized by the cells and distributed around the nuclei, demonstrating that 89WP itself has strong cellular permeability. Moreover, both siRNA-Cy5/PA/89WP and siRNA/PA/89WP-FAM were labeled with single fluorescent dye, and could be well internalized by the cells, indicating that 89WP could not only penetrate the cell membrane barrier by itself, but also facilitate the polyplexes to enter the cells. In the cells treated with double-labeled polyplex siRNA-Cy5/PA/89WP-FAM, the yellow particles were the co-localized siRNA-Cy5 and 89WP-FAM, illustrating that 89WP and siRNA co-existed in the polyplex, which could enter the cells in its entirety. The remaining green particles were free 89WP, further proving that there are not only intact polyplexes but also free 89WP in the system, as revealed by the ITC determination. For the lipoplex siRNA-Cy5/Lipo, visible cellular internalization also occurred, and the uptake efficiency was comparable to that of siRNA-Cy5/PA/89WP.

### 3.5. Cellular Uptake Pathway of siRNA Polyplexes

**Figure 6:**
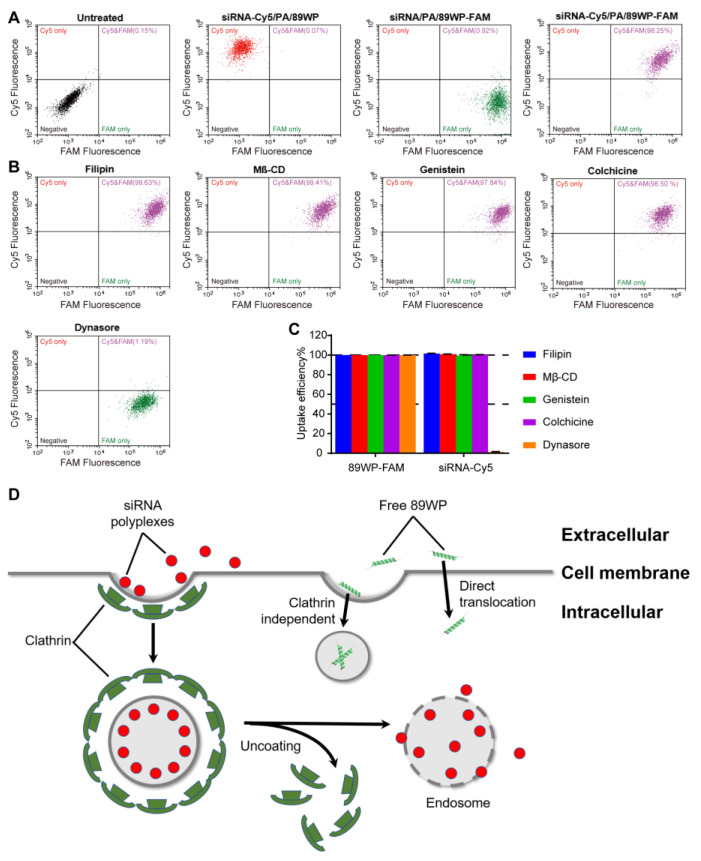
Cellular uptake pathway of siRNA polyplexes in ARPE-19 cells. (**A**) Flow cytometry diagrams of ARPE-19 cells treated with polyplexes siRNA-Cy5/PA/89WP, siRNA/PA/89WP-FAM, and siRNA-Cy5/PA/89WP-FAM, respectively. (**B**) Flow cytometry diagrams of ARPE-19 cells treated with polyplex siRNA-Cy5/PA/89WP-FAM in the presence of various cellular uptake inhibitors. (**C**) Effects of the inhibitors on the uptake efficiency of siRNA-Cy5/PA/89WP-FAM by ARPE-19 cells (*n* = 3). (**D**) Endocytosis pathway of the polyplex siRNA/PA/89WP mediated by clathrin.

Flow cytometry diagrams of WERI-Rb-1 and ARPE-19 cells internalizing siRNA polyplexes, in which siRNA was labeled with Cy5 and/or 89WP was labeled with FAM, are shown in Figure 6A and Figure S3A, respectively. In each diagram, the negative quadrant in the lower left corner represents those cells without uptake, the Cy5 quadrant in the upper left corner represents those cells internalizing siRNA-Cy5 only, the FAM quadrant in the lower right corner represents those cells internalizing 89WP-FAM only, and the Cy5 & FAM quadrant in the upper right corner represents those cells simultaneously internalizing both 89WP-FAM and siRNA-Cy5. It could be seen that the cells treated with single fluorescence-labeled polyplexes siRNA-Cy5/PA/89WP and siRNA/PA/89WP-FAM were distributed in their respective quadrants in the flow cytometry diagrams, indicating that the fluorescence signals of Cy5 and FAM dyes did not interfere with each other. In the cells treated with siRNA-Cy5/PA/89WP-FAM, the proportion of Cy5 and FAM double-positive cells reached more than 98% for ARPE-19 cells and 84% for WERI-Rb-1 cells, indicating that siRNA and 89WP almost simultaneously entered the same cells, implying that the polyplexes were internalized intactly.

The uptake efficiency was defined as the percentage of cellular uptake with an inhibitor compared to that without an inhibitor. When treated with the uptake inhibitors filipin, Mß-CD, genistein, and colchicine, no significant decrease in uptake efficiencies was observed in either ARPE-19 (Figure 6B,C) or WERI-Rb-1 (Figure S3B,C) cells, indicating that the main pathway for cellular uptake of the polyplex siRNA/PA/89WP might involve neither cholesterol- nor caveolin-mediated endocytosis nor pinocytosis. Although the uptake efficiencies of 89WP-FAM in both cells treated with siRNA-Cy5/PA/89WP-FAM remained unaffected in the presence of dynasore, those of siRNA-Cy5 were almost completely inhibited. Accordingly, the cellular uptake of polyplex siRNA/PA/89WP may be mainly via clathrin-mediated endocytosis (Figure 6D). In addition to the intact polyplex, free 89WP also existed in the delivery system, which was virtually not influenced by dynasore, implying that it was internalized via different pathways.

The uptake of fluorescence-labeled peptide 89WP-FAM and primary polyplex siRNA-Cy5/PA in ARPE-19 and WERI-Rb1 cells revealed that these inhibitors did not affect cellular uptake of the peptide alone, but that of polyplex siRNA-Cy5/PA was significantly restrained (Figure S4). This result further confirmed that the polyplexes with or without 89WP modification entered cells mainly through clathrin-mediated endocytosis, which was different from the free peptide 89WP.

### 3.6. In Vivo Retinal Distribution of siRNA Polyplexes

**Figure 7:**
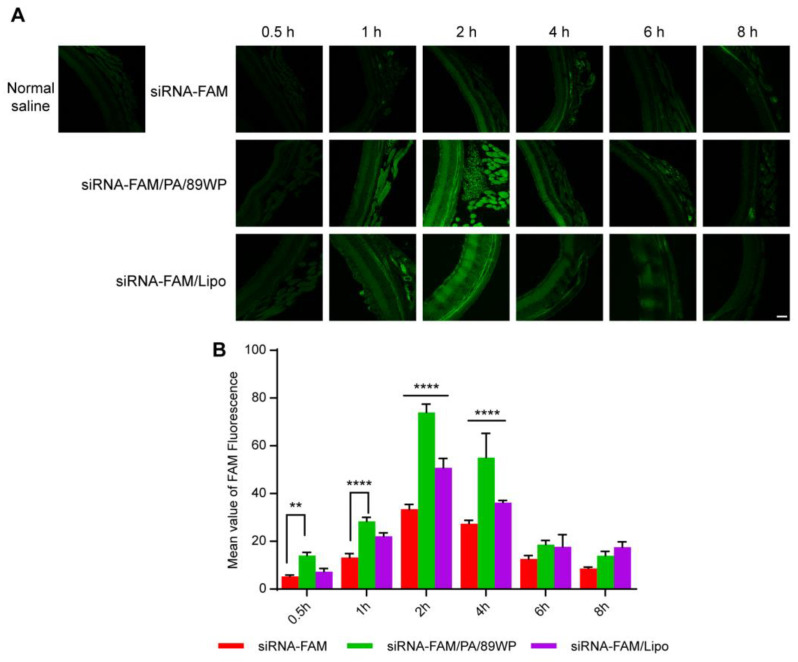
In vivo retinal distribution of siRNA formulations. All fluorescence-labeled naked siRNA-FAM, polyplex siRNA-FAM/PA/89WP, and lipoplex siRNA-FAM/Lipo contained 2 μg siRNA and were instilled into the conjunctival sac of mice. After the eyeballs were harvested at different time points, the green fluorescence intensity in the retina was observed under a confocal fluorescence microscope (**A**) and semi-quantitatively analyzed using ZEN software (**B**). Data are presented as mean ± SD (*n* = 3). Scale bar, 50 µm. Significance was compared with siRNA-FAM/PA/89WP group.

After topical instillation of the siRNA formulations labeled with FAM, the fluorescence intensity in the mouse retina was kept at a low level within 8 h in the naked siRNA group, indicating that the naked siRNA alone was difficult to absorb into the eye due to its hydrophilicity and relatively large molecular size. In contrast, the retinal fluorescence intensity of siRNA-FAM/PA/89WP and siRNA-FAM/Lipo groups increased remarkably and peaked at 2 h after administration. Then, the fluorescence intensity decreased gradually with time, and 6 h later, it was comparable with that of the siRNA-FAM group (Figure 7A). According to quantitative analysis (Figure 7B), the fluorescence intensity of siRNA-FAM/PA/89WP group was 2 to 3 times higher than that of the naked siRNA-FAM group at 0.5 h, 1 h, 2 h, and 4 h, and 1.5 times higher (*p* < 0.0001) than that of the siRNA-FAM/Lipo group at 2 h and 4 h. These results provided direct evidence that due to the ocular permeability of 89WP, siRNA polyplex could be absorbed into the eye and distributed in the retina, and the amount of siRNA accumulated in the ocular fundus was significantly higher than the lipoplex siRNA/Lipo.

### 3.7. In Vivo Gene Silencing Efficacy

**Figure 8:**
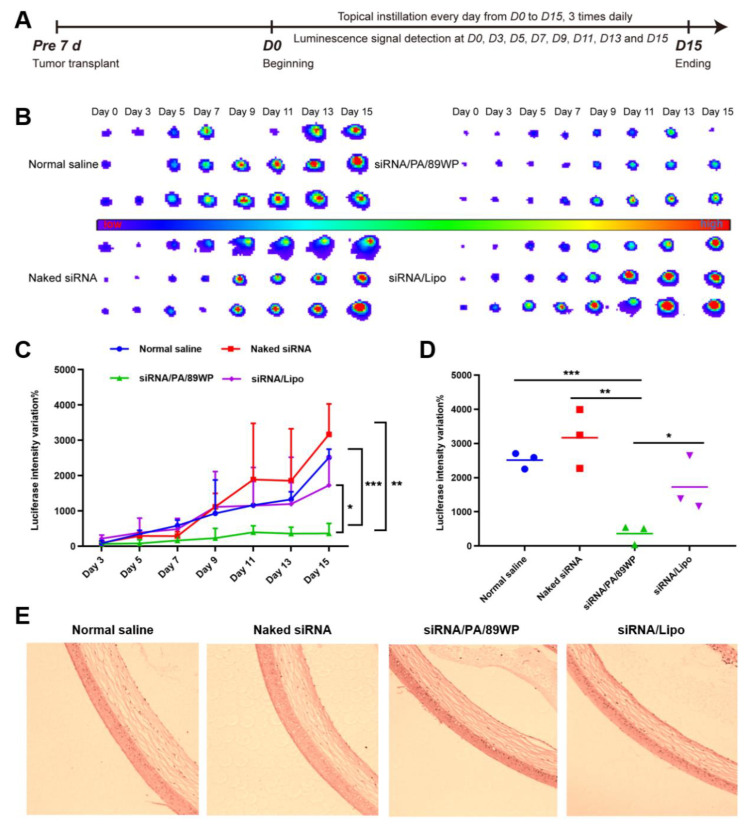
In vivo gene silencing efficacy. The eyes of nude mice were inoculated with WERI-Rb-1-luci cells, and the mice were divided into 4 groups, administered with normal saline, naked siRNA, siRNA/PA/89WP, and siRNA/Lipo, respectively. Each siRNA formulation contained 1.5 μg siRNA and was applied three times daily via topical instillation. (**A**) Time schedule of tumor transplant, treatment, and evaluation of tumor-bearing mice. (**B**) Bioluminescence imaging of the eyes. (**C**) Semi-quantitative evaluation of changes in luciferase expression. (**D**) Comparison of changes in luciferase expression on day 15. (**E**) The HE-stained cornea after administration for 15 days. Significance was compared with siRNA/PA/89WP group, and *n* = 3.

The siRNA formulations were administered as eye drops three times a day to evaluate gene silencing efficiency in retinoblastoma-bearing nude mice, and the eyes were subjected to in vivo bioluminescence imaging at set time points (Figure 8A). From Figure 8B, it could be found that the bioluminescence intensity increased significantly for the groups treated with normal saline and naked siRNA, indicating rapid proliferation of WERI-Rb-1-luci cells in the eyes. In contrast, the polyplex siRNA/PA/89WP could effectively interfere with luciferase gene expression of the intraocular WERI-Rb-1-luci cells, thereby inhibiting bioluminescence intensity at a low level similar to that at the beginning of tumorigenesis (day 0). Semi-quantitative analysis of the change in bioluminescence intensity was also conducted. The data showed that on day 15, the inhibition effect of siRNA/PA/89WP on tumor bioluminescence was 4 times better compared to that of the siRNA/Lipo group (*p* < 0.05), or 5 times and 6 times better compared to those of the groups treated with normal saline (*p* < 0.001) and naked siRNA (*p* < 0.01), respectively (Figure 8C,D). These results revealed that the polyplex siRNA/PA/89WP could efficiently penetrate across the ocular absorption barriers and delivered siRNA into the tumor cells in the posterior segment of the eye. After consecutive administration for 15 days, the corneas recovered from all the treatment groups still maintained normal morphology (Figure 8E), indicating that the siRNA formulations were safe for ocular tissues.

## 4. Discussion

Ocular gene therapy, through altering gene expression to treat genetic diseases, has great potential for a variety of degenerative retinal syndromes [29]. Currently, there are still some technical barriers in this field that urgently need to be solved, such as seeking effective therapeutic genes and developing efficient delivery vectors [19,30,31]. Although ocular gene therapy has made remarkable progress in recent years, it is still in its infancy, and vast amounts of research are in the exploratory stages. Notably, in gene delivery, the efficiency and safety of most delivery vectors cannot meet clinical requirements, which has become a technical bottleneck of gene therapy [5].

In the present study, we developed an ocular gene delivery system consisting of a cationic polymer (PAMAM) and an optimized cell-penetrating peptide (89WP). PAMAM is a synthetic dendritic polymer rich in cationic functional groups that provides a reliable binding ability with nucleic acids [32]. The binding ability increases along with the generation of dendritic molecules [33], while cytotoxicity also increases [34,35]. In order to balance the capability of gene condensation and the potential cytotoxicity caused by the vector, the 3rd generation PAMAM was selected in this work to compress siRNA and form the primary polyplex siRNA/PA by simple physical complexation.

We previously screened out penetratin from several of the most commonly reported cell-penetrating peptides based on the ex vivo permeability in excised cornea and in vivo ocular distribution [36]. In a subsequent study, we optimized wild-type penetratin via amino acid mutation to further improve its permeability, and obtained a series of derivative peptides, which could be used as potential ocular absorption enhancers with low toxicity [22]. Among these derivatives, 89WP showed more distribution in the eyes, especially in the retina, after topical instillation. Therefore, we chose 89WP to facilitate the intraocular delivery of siRNA. It seemed that the N/P ratio of 2:1 was a critical value for the 3rd generation PAMAM to compress siRNA, because the formed primary polyplex siRNA/PA exhibited a nearly neutral zeta potential (Figure 1B), revealing the negatively charged siRNA was just neutralized by PAMAM. Actually, there were still some unbound or loosely bonded siRNAs in the mixed solution, according to the results of gel electrophoresis (Figure S1). Under this circumstance, the addition of positively charged 89WP would further compress siRNA together with PAMAM, forming ternary complexes with larger particle sizes and higher zeta potential compared to siRNA/PA. To modify the polyplex with penetratin, we previously introduced low molecular weight hyaluronic acid (HA) as an electronegative linker between the positively charged polyplex and penetratin to implement layer-by-layer self-assembly via electrostatic interaction [24]. In the present work, by virtue of thermodynamic analysis, we found that 89WP and siRNA/PA could spontaneously form a polyplex without the help of HA. This finding makes the siRNA polyplex simpler in structure and easier in preparation.

Both ARPE-19 and WERI-Rb-1 are retina-related cells, and here were chosen to predict the absorption of polyplex formulations in the posterior segment of eyes. In vitro evaluations showed that the polyplexes exhibited a much stronger uptake and silencing effect in both normal cells ARPE-19 and tumor cells WERI-Rb-1. With the aid of PAMAM and 89WP, the polyplexes were able to enter the cells through the clathrin-mediated pathway. Interestingly, the internalization process of the polyplex was different from that of peptide 89WP, which was not liable to be affected by uptake inhibitors, probably due to their direct binding and interaction with negatively charged cell membranes [37,38].

The interaction between the components of polyplex siRNA/PA/89WP and its integrity after cellular uptake was also investigated. The thermodynamic parameters revealed that a strong electrostatic interaction between PA and siRNA facilitated the formation of the primary polyplex. In contrast, when 89WP was mixed with siRNA/PA, entropy-driven hydrophobic interaction played the leading role, as indicated by negative ΔG and positive ΔS [28], consequently resulting in a system containing polyplex siRNA/PA/89WP and free peptide 89WP. This could explain why 89WP-FAM was still internalized in the cells when inhibited by dynasore. The confocal results showed that siRNA overlapped with 89WP in the cells, indicating that the polyplexes were internalized as an intact form and entered the cells via the clathrin-mediated endocytosis pathway. Besides the intact polyplexes, there was some free 89WP in the formulation. Even if the internalization of polyplex siRNA/PA/89WP was suppressed by dynasore, the free 89WP might enter cells through direct bonding with a negatively charged cytomembrane.

Fluorescence-labeled polyplexes were then instilled in the eyes of the mice. We found that after 2 h, the fluorescence intensity of polyplex siRNA-FAM/PA/89WP reached the maximum in the retina, then began to gradually attenuate, and almost totally eliminated after 6 h. The ocular pharmacokinetics revealed that the eye drops of siRNA polyplexes could be absorbed into the posterior segment within a short time after administration. Consequently, bioluminescence expressed in WERI-Rb-1-luci tumor cells could be effectively inhibited by topically applied siRNA/PA/89WP, and the interference effect was significantly better than that of the lipoplex siRNA/Lipo. The tumor-inhibiting efficiency was also noninferior to the polyplex constructed with the 5th generation PAMAM and wild-type penetratin [23], which may be attributed to the improved permeability of 89WP across the ocular absorption barriers and its hydrophobic interaction with siRNA/PA. More importantly, the present polyplex was composed of lower generation PAMAM, which was favorable for reducing the potential risk of safety for ocular application. These results provided solid proof that via noninvasive administration siRNA polyplex could distribute in the fundus oculi and therefore was competent to play the role of gene therapy in related diseases.

## 5. Conclusions

In this work, we successfully constructed a noninvasive intraocular gene delivery system, in which the 3rd generation PAMAM dendrimer compressed siRNA to form the primary polyplex siRNA/PA and then the penetratin derivative 89WP was introduced to self-assemble the ternary complex system targeting fundus oculi. The formed polyplex siRNA/PA/89WP was able to enter the ocular cells in an intact form through clathrin-mediated endocytosis, and was much safer to ocular cells than the siRNA lipoplex composed of commercialized cationic liposome. When topically applied in vivo, the polyplex siRNA/PA/89WP could reach the posterior segment of eyes in a short time, and perform an interference effect on the intraocular tumor without impairing anterior ocular tissues. Therefore, this simple and safe delivery system could be a useful tool for gene therapy for hereditary intraocular diseases.

## Figures and Tables

**Table 1:** Thermodynamic parameters of siRNA binding to PAMAM and 89WP binding to siRNA/PA.

Sample	K_b_ (×10^5^ M^−1^)	ΔH (kcal/mol)	TΔS (kcal/mol)	ΔG (kcal/mol)
PA + siRNA	363 ± 125	−71.93 ± 2.02	−61.69	−10.24 ± 2.02
89WP + siRNA/PA	1.85 ± 0.90	1.04 ± 0.08	8.22	−7.18 ± 0.08

## Data Availability

All relevant data is contained in the article.

## Supplementary Materials

The following supporting information can be downloaded at: https://www.mdpi.com/article/10.3390/pharmaceutics15030745/s1. Table S1. Cellular uptake inhibitors and their function mechanisms. Figure S1. Polyacrylamide gel electrophoresis of primary polyplexes siRNA/PA. The ratio is N/P of 3rd generation PAMAM to siRNA. Figure S2. Time-dependent particle size variation of siRNA/PA/89WP incubated in 10 mM PBS at 4 °C (*n* = 3). Figure S3. Cellular uptake pathway of the siRNA polyplexes in WERI-Rb-1 cells. (A) Flow cytometry diagrams of WERI-Rb-1 cells treated with polyplexes siRNA-Cy5/PA/89WP, siRNA/PA/89WP-FAM, and siRNA-Cy5/PA/89WP-FAM, respectively. (B) Flow cytometry diagrams of WERI-Rb-1 cells treated with polyplex siRNA-Cy5/PA/89WP-FAM in the presence of various cellular uptake inhibitors. (C) Effects of the inhibitors on uptake efficiency of siRNA-Cy5/PA/89WP-FAM by WERI-Rb-1 cells (*n* = 3). Figure S4. Effects of various inhibitors on uptake efficiency of single fluorescence-labeled peptide (89WP-FAM) and primary polyplex (siRNA-Cy5/PA) in ARPE-19 (A) and WERI-Rb-1 (B) cells (*n* = 3).
