# Supplementary material for: Intraocular siRNA Delivery Mediated by Penetratin Derivative to Silence Orthotopic Retinoblastoma Gene
Source: Pharmaceutics. 2023 Feb 23;15(3):745. doi: 10.3390/pharmaceutics15030745 (PMC10053059; doi:10.3390/pharmaceutics15030745)
Supplement: Supplementary file 1 [file pharmaceutics-15-00745-s001.zip › pharmaceutics-2037790-supplementary.pdf]

Intraocular siRNA Delivery Mediated by Penetratin Derivative to Silence Orthotopic Retinoblastoma Gene

Xin Gao [1,†], Xingyan Fan [1,†], Kuan Jiang [1,2], Yang Hu [1], Yu Liu [1], Weiyue Lu [1,3], Gang Wei [1,3,4,*]

[1] Key Laboratory of Smart Drug Delivery, Ministry of Education & Department of Pharmaceutics, School of Pharmacy, Fudan University, Shanghai 201203, China

[2] Department of Pharmacology, School of Basic Medical Sciences & State Key Laboratory of Molecular
Engineering of Polymers, Fudan University, Shanghai 200032, China

[3] The Institutes of Integrative Medicine of Fudan University, Shanghai 200040, China

[4] Shanghai Engineering Research Center of ImmunoTherapeutics, Shanghai 201203, China

***** Correspondence: weigang@shmu.edu.cn; Tel.: +86-21-5198-0091; Fax: +86-21-5198-0090

† These authors contributed equally to this work.

Table S1. Cellular uptake inhibitors and their function mechanisms

Inhibitor	Final concentration	Mechanism of action
Filipin	20 μg/mL	Bind to cholesterol on the cell membrane and inhibit endocytosis
Mß-CD	300 μg/mL	Deplete cholesterol and suppress endocytosis
Genistein	20 μg/mL	Inhibit lipid raft/caveolae mediated endocytosis
Colchicine	20 μg/mL	Inhibit pinocytosis by binding to tubulin to prevent microtubule polymerization
Dynasore	20 μg/mL	Inhibit dynamic proteins in clathrin-mediated endocytosis

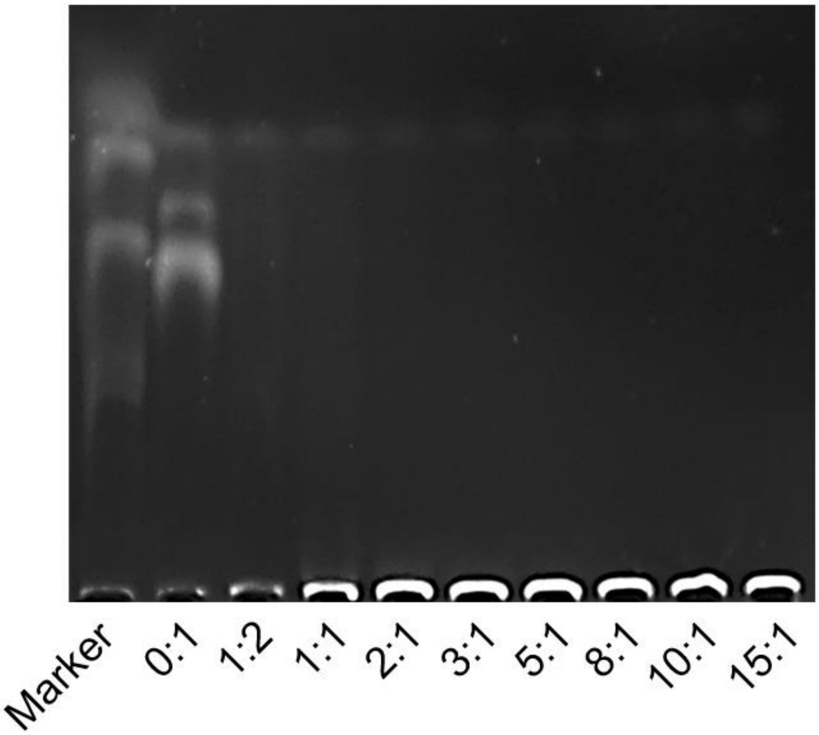

Figure S1. Polyacrylamide gel electrophoresis of primary polyplexes siRNA/PA. The ratio is N/P of 3rd generation PAMAM to siRNA.

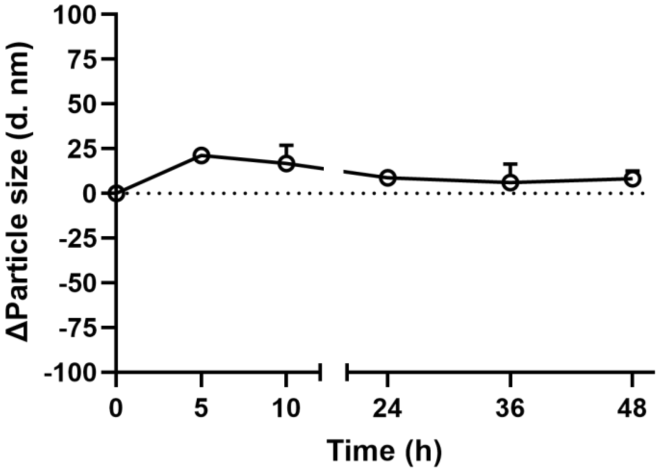

Figure S2. Time-dependent particle size variation of siRNA/PA/89WP incubated in 10 mM PBS at 4 °C (*n*=3).

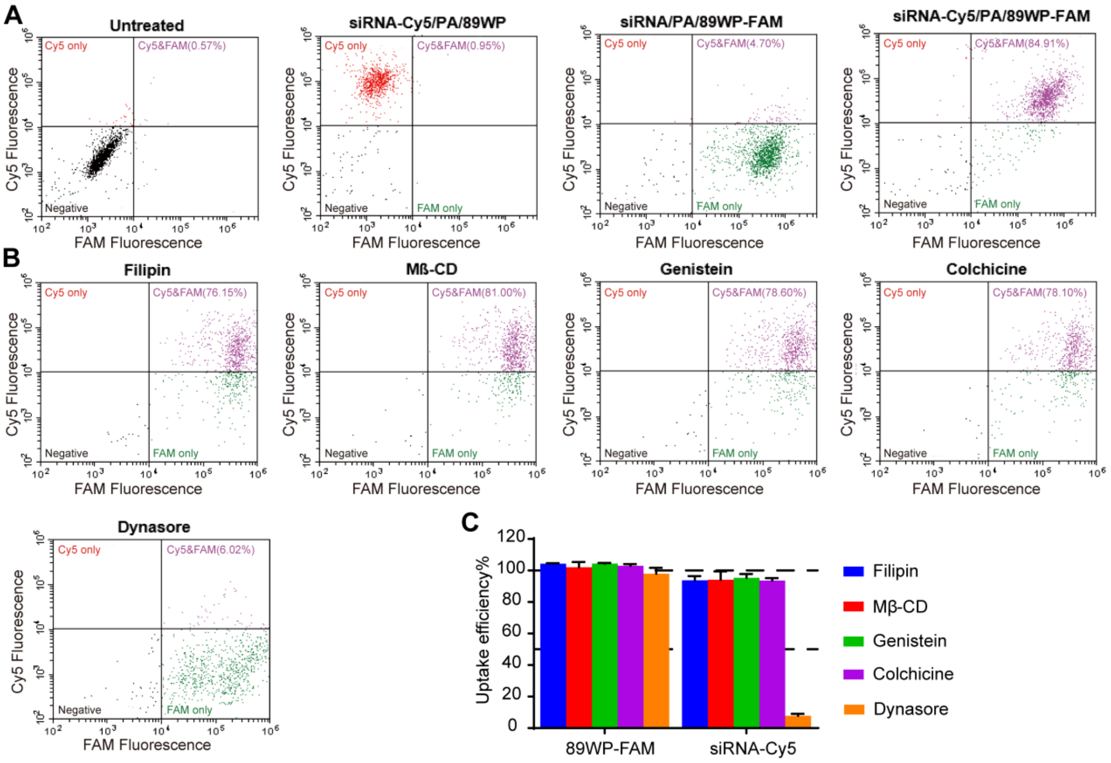

Figure S3. Cellular uptake pathway of the siRNA polyplexes in WERI-Rb-1 cells. (A) Flow cytometry diagrams of WERI-Rb-1 cells treated with polyplexes siRNA-Cy5/PA/89WP, siRNA/PA/89WP-FAM, and siRNA-Cy5/PA/89WP-FAM, respectively. (B) Flow cytometry diagrams of WERI-Rb-1 cells treated with polyplex siRNA-Cy5/PA/89WP-FAM in the presence of various cellular uptake inhibitors. (C) Effects of the inhibitors on uptake efficiency of siRNA-Cy5/PA/89WP-FAM by WERI-Rb-1 cells (*n*=3).

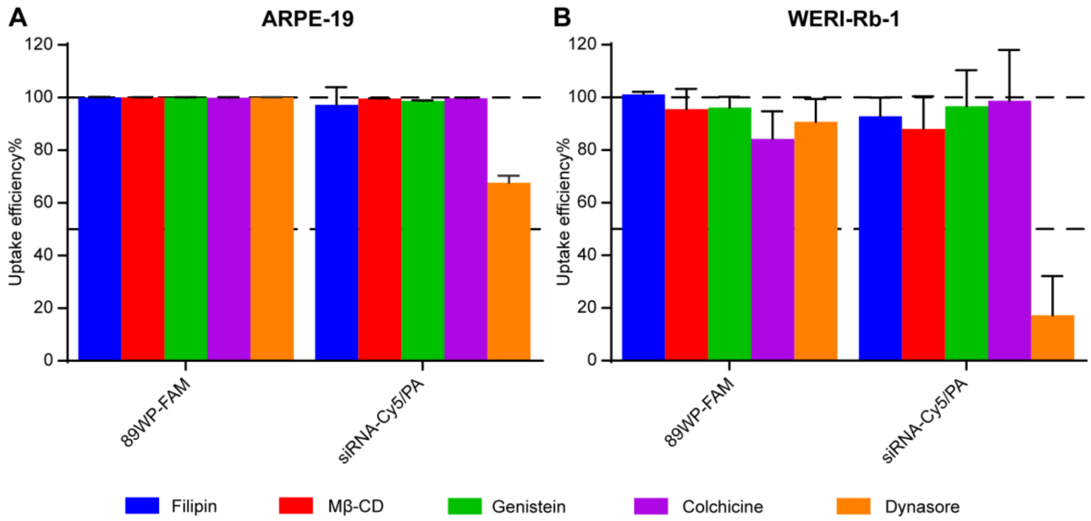

Figure S4. Effects of various inhibitors on uptake efficiency of single fluorescence-labeled peptide (89WP-FAM) and primary polyplex (siRNA-Cy5/PA) in ARPE-19 (A) and WERI-Rb-1 (B) cells (*n*=3).
